# Supplementary material for: The Systems Biology Research Tool: evolvable open-source software
Source: BMC Syst Biol. 2008 Jun 29;2:55. doi: 10.1186/1752-0509-2-55 (PMC2446383; doi:10.1186/1752-0509-2-55)
Supplement: Additional file 1 — SBRT Archive. An archive of the current version of the Systems Biology Research Tool. [file 1752-0509-2-55-S1.zip › sbrt-1.4.0/doc/developers_guide/api/sbrt/shell/mng/fba/package-summary.html]

sbrt.shell.mng.fba


|  |  |  |
| --- | --- | --- |
| **Overview** \| **Package** \| Class \| **Use** \| **Tree** \| **Deprecated** \| **Index** \| **Help** | |  |
| **PREV PACKAGE**   **NEXT PACKAGE** | **FRAMES**    **NO FRAMES**     **All Classes** |  |


---

## Package sbrt.shell.mng.fba

Provides classes and interfaces for creating Flux Balance Analysis
process managers.

**See:**
  
          **Description**

| **Class Summary** | |
| --- | --- |
| **BiggSbmlReaderManager** | This class is used to manage the reading of SBML files obtained from the BiGG Database. |
| **BiggSbmlTranslaterManager** | This class is used to manage the translation of SBML files from the BiGG Database into the Systems Biology Research Tool's *FBA Reaction Files* and *FBA Catalyst Files*. |
| **CatalystDeletionManager** | This class is used to manage FBA catalyst deletion experiments. |
| **CdHarFluxVectorManager** | This class is used to manage FBA coordinate direction hit-and-run random flux vector experiments. |
| **CvCatalystDeletionManager** | This class is used to manage FBA constraint variation-catalyst deletion experiments. |
| **CvFbaOptManager** | This class is used to manage constraint variation-FBA optimization processes. |
| **CvMsFluxIntervalManager** | This class is used to manage constraint variation-Mahadevan-Schilling flux interval experiments. |
| **CvObjectiveFunctionManager** | This class is used to manage FBA constraint variation-objective function experiments. |
| **CvRevRxnFluxIntervalManager** | This class is used to manage constraint variation-constrained reverse reaction flux interval experiments. |
| **CvRxnDeletionManager** | This class is used to manage FBA constraint variation-reaction deletion experiments. |
| **CvSimpleFluxIntervalManager** | This class is used to manage constraint variation-simple flux interval experiments. |
| **ExtremeCurrentManager** | This class is used to manage FBA extreme current experiments. |
| **ExtremeCurrentManager.Rm** |  |
| **FbaManager** | This abstract class provides a foundation for Flux Balance Analysis managers. |
| **FbaOptManager** | This class is used to manage basic FBA optimization experiments. |
| **FbaSolverManager** | This class is used to manage FBA solver processes. |
| **FluxActivityManager** | This class is used to manage flux activity experiments. |
| **FluxCapManager** | This class is used to manage flux cap experiments. |
| **FluxPlasticityManager** | This class is used to manage flux plasticity experiments. |
| **InitialPointManager** | This class is used to manage initial point experiments. |
| **IntervalVariationManager** | This abstract class provides utitlity functions for managing flux interval variation experiments. |
| **MetatoolFileWriterManager** | This class is used to manage the writing of input files for Metatool. |
| **MmaNotebookManager** |  |
| **MsFluxIntervalManager** | This class is used to manage Mahadevan-Schilling flux interval experiments. |
| **MsFluxomeReducerManager** | This class is used to manage the MS fluxome reduction algorithm. |
| **NetworkInfoManager** | This class is used to manage fluxome information experiments. |
| **ObjectiveFunctionManager** | This class is used to manage objective function experiments. |
| **PalssonSbmlReaderManager** | This class is used to manage the reading of SBML files obtained from Palsson's website. |
| **PalssonSbmlTranslaterManager** | This class is used to manage the translation of SBML files from Palsson's website into the Systems Biology Research Tool's *FBA Reaction Files* and *FBA Catalyst Files*. |
| **RandomConstraintsManager** | This class is used to manage random constraints generators. |
| **RandomEnvManager** | This class is used to manage random environment generators. |
| **RandomFluxVectorManager** | This abstract class provides a foundation for random flux vector managers. |
| **RandomObjectiveManager** | This class is used to manage random objective function generators. |
| **ReverseRxnFluxIntervalManager** | This class is used to manage constrained-reverse reaction flux interval experiments. |
| **RxnDeletionManager** | This class is used to manage FBA reaction deletion experiments. |
| **RxnRemovalManager** | This class is used to manage the removal of reactions from stoichiometric networks. |
| **SbmlWriterManager** | This class is used to manage the translation of Systems Biology Markup Language (SBML) files into Systems Biology Research Tool (SBRT) Fluxome Files. |
| **SimpleFluxIntervalManager** | This class is used to manage simple flux interval experiments. |
| **SimpleRxnFileTransManager** | This class is used to manage the translation of simple reaction files files into Systems Biology Research Tool (SBRT) FBA Reaction Files. |
| **SlpCycleIdManager** | This class is used to manage steady state cycle identification. |
| **StoichVariationManager** | This class is used to manage stoichiometry variation-FBA optimization processes. |
| **SvCdHarFluxVectorManager** | This class is used to manage flux space variation, coordinate direction hit-and-run random flux vector experiments. |
| **SvInitialPointManager** | This class is used to manage flux space variation-initial point experiments. |
| **WwFluxomeReducerManager** | This class is used to manage the WW fluxome reduction algorithm. |

## Package sbrt.shell.mng.fba Description

Provides classes and interfaces for creating Flux Balance Analysis
process managers.

---


|  |  |  |
| --- | --- | --- |
| **Overview** \| **Package** \| Class \| **Use** \| **Tree** \| **Deprecated** \| **Index** \| **Help** | |  |
| **PREV PACKAGE**   **NEXT PACKAGE** | **FRAMES**    **NO FRAMES**     **All Classes** |  |


---
